# Neurological Manifestations of Chronic Methadone Maintenance Therapy: A Case Report and Literature Review

**DOI:** 10.7759/cureus.29534

**Published:** 2022-09-24

**Authors:** Ulviyya Gasimova, Khurram M Afzal, Aninda B Acharya

**Affiliations:** 1 Neurology, Saint Louis University School of Medicine, St. Louis, USA

**Keywords:** toxicology, leukoencephalopathy, choreiform movements, basal ganglia, methadone

## Abstract

Methadone is a long-acting opioid medication that is used as maintenance therapy for heroin addiction. We present a case of a patient on methadone maintenance therapy for chronic back pain who developed neurological complications. The patient presented with mental status changes and choreiform movements. Workup revealed lesions involving the subcortical white matter and basal ganglia. Choreiform movements improved after the initiation of treatment with topiramate, clonazepam, and risperidone. This combination was chosen as several prior case reports published significant benefit and improvement in choreiform movements with the mentioned regimen.

## Introduction

Methadone (buprenorphine) is a synthetic long-acting opioid that is often used to treat heroin addiction and chronic pain [1]. Methadone is lipid-soluble and is known to act upon the limbic system and cerebellum, which have a high density of mu-opioid receptors [2-4]. Although it binds to the same receptors as other opioids, it does so more slowly and is less likely to lead to a euphoric sensation. As a result, methadone alleviates withdrawal and craving symptoms common in addiction to heroin and other opioids. Maintenance therapy with methadone due to uptake by brain tissues in the long term may affect neuromechanisms. The mechanism is mainly thought to be via dysregulation of gliogenesis [5].

## Case presentation

This is a case of a 40-year-old man who was independent at baseline. He was admitted to an outside hospital (OSH) with reported altered mental status. His medical history was notable for chronic back pain for which he was on long-standing (>5 years) methadone maintenance therapy. Comorbidities included chronic hepatitis C status post-treatment with a direct-acting antiviral medication years ago, as well as depression. Home medications included ferrous gluconate, fluticasone and salmeterol inhalers, aspirin, and methadone. He lived with his wife and daughter and was able to perform all his activities of daily life. His family reports that he had no premorbid cognitive impairment. The patient had no history of alcohol or drug use and quit smoking nine years ago after smoking two packs a day for 15 years.

He was in his usual state of health until a month ago prior to admission to an OSH. He awoke the morning of presentation to the OSH with altered level of consciousness, bilateral leg weakness, and urine incontinence. Workup at the OSH was notable for positive coronavirus variant 229E. Computed tomography (CT) of the chest, abdomen, and pelvis revealed patchy bilateral airspace infiltrates with scattered mediastinal lymph nodes, nonspecific bilateral punctate non-obstructing renal calyceal stones, and a small left inguinal hernia. Head CT was unremarkable. Magnetic resonance imaging (MRI) of the brain showed bilateral multiple small areas of deep white matter infarctions including left basal ganglia, globus pallidus, and bilateral subcortical white matter. His presentation was thought to be a result of metabolic or infectious etiology. He was discharged home with doxycycline, cefdinir, and atorvastatin. After discharge, his mental status and strength improved, but he did not return to his baseline.

Three days post-discharge, he had a recurrence of his decreased level of consciousness and was again admitted to the OSH. At the time, he was noted to have moderately increased creatinine levels. The repeated head CT did not reveal acute changes. He was then transferred to our institution.

On admission to our hospital, he was oriented to himself only, but not to place, date, or situation. He had non-fluent speech. The motor examination showed weakness and increased muscle tone in both legs. With arms held out, he was noted to have asterixis in both arms, more prominent on the right side. Muscle stretch reflexes showed increased reflexes in the lower extremity, as well as upgoing toes and sustained clonus bilaterally. Because of his mental status, the sensory examination was unreliable.

**Figure 1:**
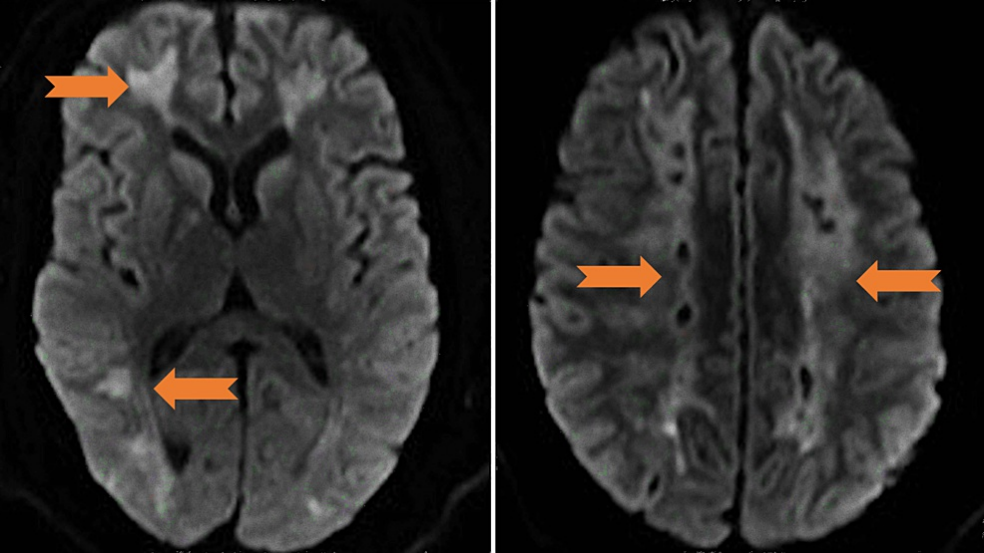
DWI sequence with mild restricted diffusion and patchy non-mass-like enhancement at bilateral subcortical regions without mass effect (arrows). DWI: diffusion-weighted imaging

**Figure 2:**
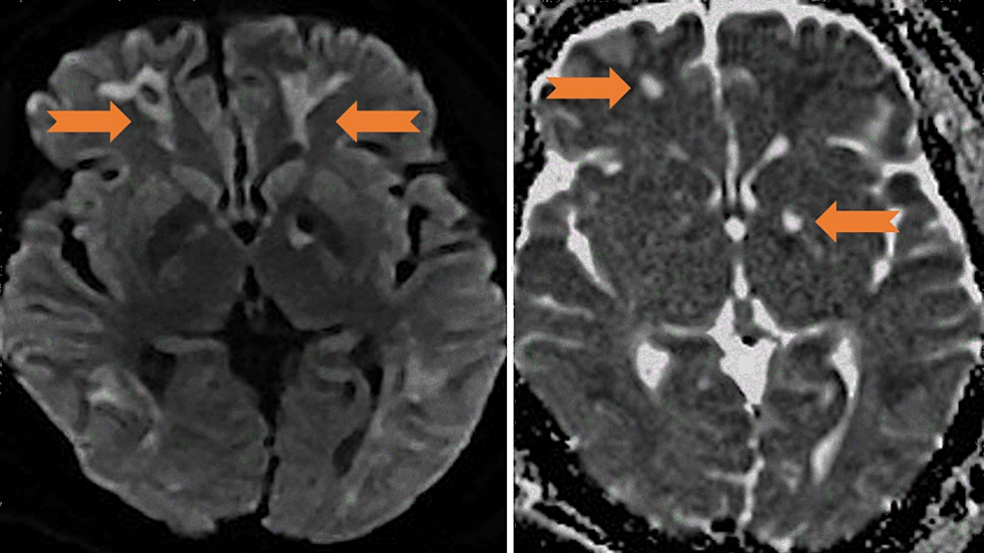
DWI (left) and ADC (right) with basal ganglia, left more than right diffusion restriction with ADC correlate (arrows). DWI: diffusion-weighted imaging; ADC: apparent diffusion coefficient

**Figure 3:**
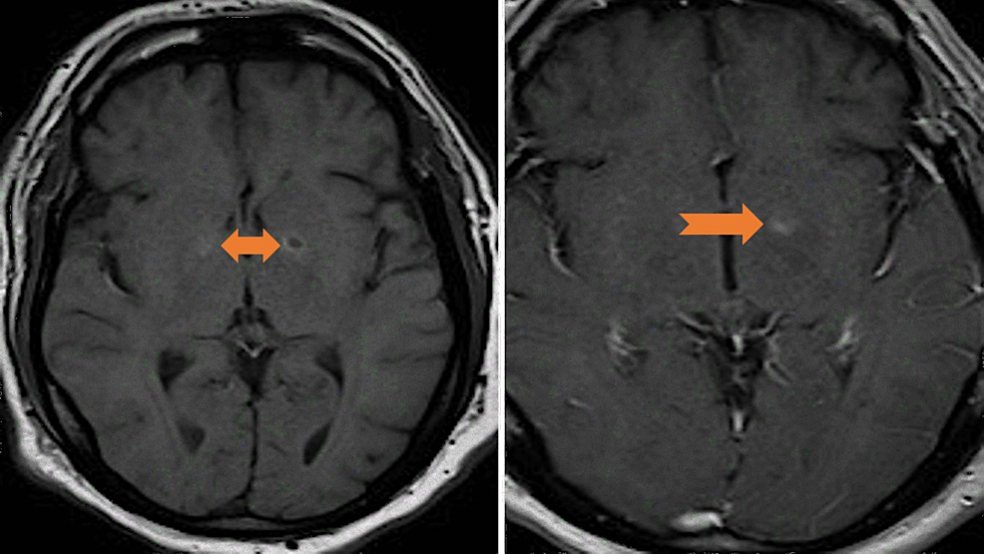
MRI of the brain T1 sequence axial images with and without contrast. Mild restricted diffusion and patchy non-mass-like enhancement in portions of this signal abnormality without mass effect (arrows). MRI: magnetic resonance imaging

**Figure 4:**
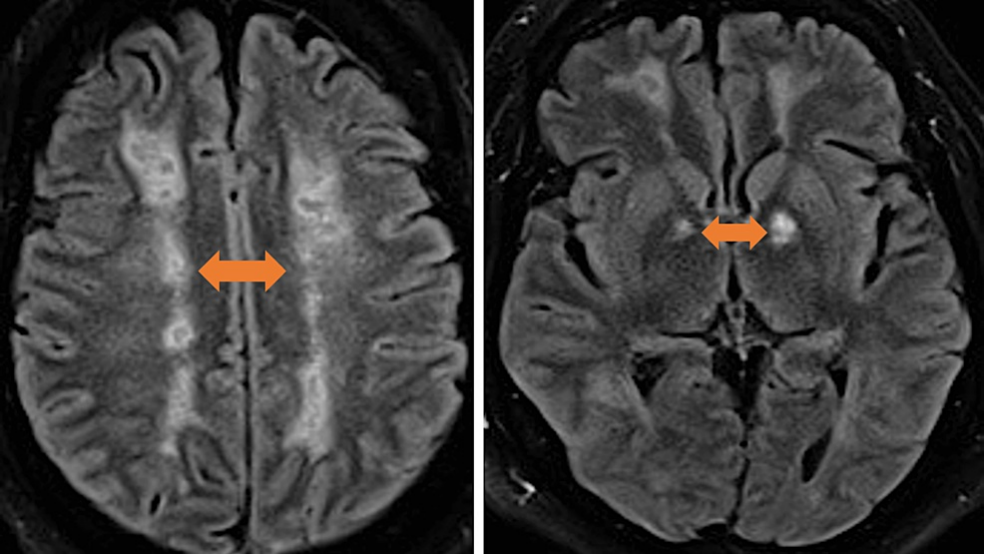
MRI of the brain T2/FLAIR sequence axial images with confluent white matter signal abnormality in bilateral frontoparietal and occipital white matter. Multiple small areas of cystic encephalomalacia in bilateral frontal and parietal deep white matter and bilateral globus pallidus (arrows). MRI: magnetic resonance imaging; FLAIR: fluid-attenuated inversion recovery

On hospital day 3, he was noted to have choreiform movements in his right arm and leg. Laboratory workup revealed a mildly elevated creatine kinase (CK). The urine drug screen was positive for methadone, but no other opioids. Further workup for metabolic, infectious, or inflammatory causes was unremarkable. ​Brain MRI with and without contrast was obtained, and the findings are discussed in Figures 1, 2, 3, 4.

Lumbar puncture with cerebrospinal fluid analysis for cell count, protein, glucose, infectious factors, cytology, autoimmune/inflammatory, and paraneoplastic workup was unrevealing. Continuous electroencephalogram (EEG) noted generalized slowing consistent with encephalopathy with no electrographic seizures. MRI of the cervical, thoracic, and lumbar spine did not show any significant abnormalities. For the choreiform movements, the patient was started on a regimen of topiramate 50 mg twice daily, clonazepam 0.5 mg thrice daily, and risperidone 0.5 mg at bedtime. The effect of topiramate on choreiform movements is associated with its effect on gamma-aminobutyric acid (GABA) activity and the modulation of voltage-gated ion channels, while risperidone works via the reduction dopaminergic pathway. This helped the abnormal movements significantly, and he was discharged on this regimen to a rehabilitation facility.

## Discussion

Prior studies investigated the neurotoxic effects of methadone on the central nervous system. In these studies, MRI findings were consistent with symmetric hyperintensities involving the cerebellum, basal ganglia, corona radiata, subcortical U fibers, and subcortical white matter [6-9]. Li et al. investigated the neurological consequences of chronic methadone consumption using diffusion tensor imaging. This group reported significantly decreased fractional anisotropy and axial diffusivity with increased radial diffusivity in extensive white matter regions alongside the involvement of the left posterior limb and internal capsule, superior and posterior corona radiata, bilateral external capsule, and right superior longitudinal fasciculus suggesting the neurotoxic effect of methadone in white matter tissues of the brain. Methadone toxicity has been reported to lead to cognitive dysfunction including alterations in memory, attention, visuospatial analysis, learning, and executive function [10-12].

Our patient presented with decreased level of consciousness, impaired attention, orientation, language, and memory. MRI revealed lesions in the cerebral white matter and basal ganglia. He had signs and symptoms consistent with pyramidal and extrapyramidal tract involvement.

## Conclusions

Methadone is widely used in patients with heroin addiction, as well as in populations with chronic pain. However, in some cases, the risks and benefits of methadone usage are controversial. This case report highlights that methadone use can lead to extensive injury to the central nervous system, mostly involving white matter and basal ganglia. Long-term methadone use and its possible consequences should be discussed thoroughly with patients and family members.
